# *Rhamphempis*, a New Genus of Empidini (Diptera: Empididae: Empidinae) of the New World, with Descriptions of Five New Species from French Guiana and the Eastern United States

**DOI:** 10.3390/insects15070524

**Published:** 2024-07-11

**Authors:** Christophe Daugeron, José Albertino Rafael, Dayse W. A. Marques

**Affiliations:** 1 Muséum National d’Histoire Naturelle, Centre National de la Recherche Scientifique, Mécanismes Adaptatifs et Évolution, UMR 7179 MNHN-CNRS MECADEV, CP 50, 45 rue Buffon, 75005 Paris, France; 2 Instituto Nacional de Pesquisas da Amazônia, INPA, Coordenação de Biodiversidade, Manaus 69060-000, AM, Brazil; jarafael@inpa.gov.br (J.A.R.); willkenia@gmail.com (D.W.A.M.)

**Keywords:** new genus, new species, Neotropical and Nearctic regions, French Guiana, USA

## Abstract

**Simple Summary:**

A new genus of empidine dance fly, namely *Rhamphempis*, is described with five new species included. The new genus is recorded from French Guiana and the United States of America. The peculiar disjunct geographic distribution of *Rhamphempis* is discussed.

**Abstract:**

The genus *Rhamphempis* **gen. nov.** (Diptera: Empididae: Empidinae: Empidini) is described and includes the following five new species from French Guiana and the USA: *Rhamphempis concava* **sp. nov.** (France: French Guiana, Roura); *R. distincta* **sp. nov.** (France: French Guiana, Roura); *R. mirifica* **sp. nov.** (France: French Guiana, Régina); *R. montreuili* **sp. nov.** (Type species, France: French Guiana, Mitaraka, Roura, St-Georges-de-l’Oyapock); and *R. septentrionalis* **sp. nov.** (USA: Maryland, College Park). The genus differs from other empidine genera by the following combination of characters: scape and postpedicel lengthened, proboscis long, strongly sclerotised with labella as long as prementum bearing annulations, wing with R_2+3_ somewhat shortened, more or less recurved at pterostigma, R_4+5_ unforked, base of abdomen yellowish in male, brownish to blackish in female, male pregenital segments strongly modified and postabdomen more or less downcurved, presence of large surstylus, very fine and long phallus. The genus is fully illustrated and keyed along with a discussion of its peculiar disjunct geographic distribution and its phylogenetic relationship within the tribe Empidini.

## 1. Introduction

Among the family Empididae (Diptera, Empidoidea), the subfamily Empidinae is the most diverse and includes more than 2000 known species (Yang et al. (2007) [1]), although thousands of others undoubtedly have yet to be described (e.g., see Bickel (1996) [2]).

The Empidinae colonized all temperate or cold areas of the planet (the whole Holarctic realm, Patagonia, South Africa, southwest and southeast Australia, including Tasmania, and New Zealand) and most mountain ranges, including those in tropical areas. Some species are even known from the Arctic Basin (e.g., see Sinclair et al. (2019) [3]). However, there are some species groups that have also considerably diversified in tropical lowland forests in the Amazon basin, tropical Africa, or Southeast Asia (e.g., see Rafael & Ale-Rocha (2002) [4]; Daugeron & Grootaert (2003) [5]).

The Empidinae are traditionally divided into two tribes, Empidini and Hilarini. The Empidini are distinguished from the Hilarini by a setulose laterotergite, enlarged male cerci, costal wing vein ending, or weakened, beyond the radial vein, and vein R_1_ not swollen apically (Sinclair & Cumming (2006) [6]).

Like Hilarini, Empidini displays an extraordinary range of mating behaviours often unique within the animal kingdom with males producing nuptial gifts transferred to females in more or less complex swarms (e.g., see Tréhen (1971) [7], Chvála (1976) [8], Funk and Tallamy (2000) [9], Sadowski et al. (1999) [10], and Murray et al. (2022) [11] for a complete review of the mating behaviours of the Empidinae). Although in such cases, mate choice is the prerogative of females and hence conforms to current models of mate selection in insects (Alcock and Gwynne (1991) [12]), sex role-reversed mate selection is also common and appears to correlate with the presence of secondary sexual characteristics in females (Cumming (1994) [13]; Svensson & Petersson (1987) [14]). Empidini are often flower visitors, and feeding on nectar becomes obligatory for many species. They visit a wide variety of flowers belonging mainly to the Asteraceae and Rosaceae, and recent studies show that Empidini are among the main flower visitors in some environments and are, therefore, potentially the main pollinators and a key element in plant–insect interactions in ecosystems where they are abundant (Lefebvre et al. (2018) [15]).

Most species of Empidini are included in the diverse genera *Empis* Linnaeus, 1758, and *Rhamphomyia* Meigen, 1822, which include together about 1300 species (Yang et al. (2007) [1]), which are divided into subgenera originally based on Bezzi (1909) [16], with some of them now considered paraphyletic (Daugeron et al. (2011) [17]). Both genera are mainly distributed in the northern hemisphere, whereas many southern species of *Empis*, especially described from temperate areas of South America, are probably not congeneric and could even be more closely related to the tribe Hilarini or be basal to the subfamily. In addition to these two highly diversified genera, the Empidini includes 12 additional genera, for which we give here the distribution only when at least one species has been described: *Bolrhamphomyia* Rafael, 2010 (one species, Neotropical), *Chilerhamphomyia* Rafael, 2010 (one species, Neotropical), *Clinorhampha* Collin, 1933 (three species, Neotropical), *Edenophorus* Smith, 1969 (seven species, Afrotropical), *Empidadelpha* Collin, 1928 (three species, Neotropical, Australasian), *Hystrichonotus* Collin, 1933 (one species, Neotropical), *Lamprempis* Wheeler & Melander, 1901 (25 species, Neotropical), *Macrostomus* Wiedemann, 1817 (42 species, Neotropical), *Opeatocerata* Melander, 1928 (21 species, Neotropical), *Porphyrochroa* Melander, 1928 (54 species, Neotropical), *Rhamphella* Malloch, 1930 (one species, Australia), and *Sphicosa* Philippi, 1865 (nine species, Neotropical).

The only phylogenetic analyses of the Empidinae carried out to date, although provisional (e.g., see Daugeron et al. (2010) [18]; Winkler et al. (2010) [19]; Daugeron et al. (2011) [17]; and Watts et al. (2016) [20]), have revealed several major lineages within the Empidini. One of these, called the *Empis* clade or *Empis* lineage, includes groups of species that have a long, heavily sclerotised proboscis and particular wing characteristics (discal cell truncate, radial fork R_4+5_ at right angle). It has been shown that the Neotropical genera, which have diversified in tropical lowland forests, especially in the Amazon basin (i.e., *Lamprempis*, *Macrostomus*, *Opeatocerata,* and *Porphyrochroa*), probably form a monophyletic group rooted in this lineage, although their proboscis is rather short and thick. About 90 species of *Empis* s.lat. and *Rhamphomyia* s.lat. were described from the Neotropics and represent diverse monophyletic groups and, therefore, potential new genera to describe.

Among them, there is a very characteristic group of species, which have rings on the labellum, a strong modification of male pregenital segments and a considerable development of the surstylus. This group is particularly diversified in the Amazon rainforest but presents a curious disjunct distribution with one species distributed in eastern North America (USA, Maryland).

The main purpose of this paper is to describe this distinctive new genus of Empidini, namely *Rhamphempis* **gen. nov.**, as well as four new species from French Guiana and the unique species presently known from a single locality in eastern North America (USA, Maryland) and not collected again since the 1930s.

## 2. Materials and Methods

This study is based on specimens that will be deposited in the following institutions: the Invertebrate Collection of the Instituto Nacional de Pesquisas da Amazônia (INPA, Manaus), the Terrestrial Arthropods Collection of the Muséum national d’Histoire naturelle (MNHN, Paris), and the U.S. National Museum of Natural History (USNM, Smithsonian, Washington). An inventory number starting with the initials ED (meaning Entomology and Diptera, respectively) was attached to each specimen deposited in MNHN and data captured in the related collection database.

Species are described according to the regions where they occur (starting with the Nearctic region) and then in alphabetical order.

The morphological terminology follows McAlpine (1981) [21], Cumming and Wood (2017) [22], and Stuckenberg (1999) [23] for the antennal structure. Male genitalia were dissected and macerated in hot 10% KOH, positioned in glycerine, and drawn using a camera lucida. After study and illustration, the dissected terminalia were placed in a microvial with glycerin and then mounted below the specimen on the same pin.

The distribution maps of new species were created using SimpleMappr (Shorthouse (2010) [24]).

The photographs were taken using a Canon EOS 6D camera fitted onto a StackShot connected to a computer via software Helicon Remote. Multiple serial layers of photographs were combined into a single fully focused image with software Helicon Focus.

Label data for the same specimen, but from different labels, were separated by quotation marks (“”). The original label data were given verbatim for the type specimens. Square brackets ([ ]) were used to indicate complementary data not included on the labels.

## 3. Taxonomy

### 3.1. Rhamphempis ***gen. nov.***

urn:lsid:zoobank.org:act:8ED6D149-EBDC-4716-A041-11AC742F32BA

Type species: *Rhamphempis montreuili* **sp. nov.**

Gender: feminine

**Etymology:** The genus name is the contraction of *rhamph-* and *empis*, in reference to the two large well-known genera of Empidinae, *Rhamphomyia* (a combination of the two Greek words, *rhamphos* for bird’s beak and *myia* for fly) and *Empis* (an established generic name originally Greek for mosquito or gnat), respectively. The vein R_4+5_ is simple like in *Rhamphomyia,* and the proboscis is elongated, fine, and strongly sclerotised like in many species of the genus *Empis*.

**Diagnosis:** *Rhamphempis* belongs to the tribe Empidini; it can be distinguished from all other genera of Empidinae by the combination of the following characters: scape and postpedicel lengthened, proboscis rather long, strongly sclerotised with labella as long as prementum bearing annulations, wing with R_2+3_ somewhat shortened, more or less recurved at pterostigma, R_4+5_ unforked, base of abdomen yellowish in male, brownish to blackish in female, male pregenital segments strongly modified and male postabdomen more or less downcurved, presence of large surstylus, and very fine and long phallus.

The modifications of male pregenital segments, the surstylus-like extension of the epandrium and the shape of R_2+3_, are apomorphic characters.

**Figure 1:**
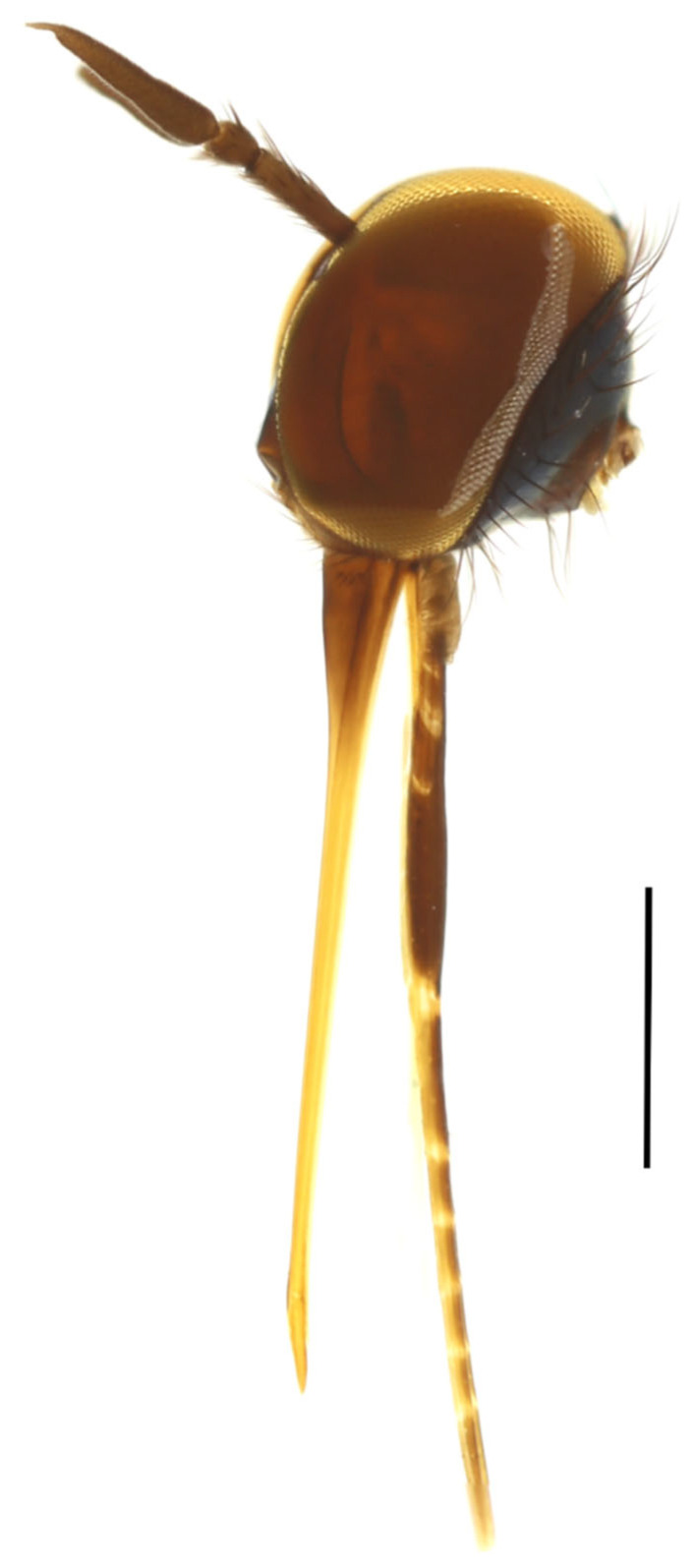
Head of *Rhamphempis montreuili* **sp. nov.** showing the annulations on the labium. Scale bar = 0.5 mm.

Annulations found in *Rhamphempis*, consisting of sclerotised plates distinctly separated by membranous parts (Figure 1), do not seem to be homologous to those found in some Afrotropical species groups (jointed sclerotised plates, see Daugeron & Grootaert (2003) [5]), so that this character is provisionally considered an additional synapomorphy for *Rhamphempis*.

The female of only two species is known; however, the unusual dimorphic colouration of the base of the abdomen (yellowish in male, brownish in female) could be an additional apomorphic character for this new genus.

**Description: Male.** Species of middle size (wing length between 3 and 4.5 mm), brownish to blackish in ground colour. *Head* with occiput blackish with row of distinct postocular setae. Ocellar triangle prominent with pair of rather short, fine setae. Frons reduced, face wide, bare. Eyes holoptic with upper ommatidia more or less enlarged. Antenna blackish, scape distinctly longer than pedicel, postpedicel elongated, stylus short, scape and pedicel with short bristly hairs. Labrum longer than head height, labella at least as long as prementum, distinctly annulated, strongly sclerotised, with a few minute, almost not visible hairs; palpus small, curved with a few fine, short setae. *Thorax* brownish to blackish in ground colour. All setae black. Antepronotum with a few setae on each side. Postpronotum with one distinct seta. Proepisternum and prosternum with at least one distinct seta. Acrostichals at least irregularly biserial. Dorsocentrals ending in one strong, very long seta in prescutellar depression. Other strong, long setae as follows: one pre-, two stronger, longer postsutural supra-alars, one postalar, three notopleurals. Laterotergite with fan of strong, very long setae. Scutellum with at least pair of strong, long apical setae. *Wing* with strong, long costal seta. R_2+3_ shortened, more or less recurved at pterostigma. Distinct brown pterostigma. Discal cell short, not truncate. Halter with brownish to blackish knob in ground colour. *Legs:* Femora and tibiae with specific arrangement of setae. *Abdomen* blackish except for brownish to yellowish base, covered with distinct not very long black setae. Tergite 6 elongated posteriorly with rounded posterior margin, sternite 6 reduced, more or less membranous, tergite 7 elongated ventrally so that segments seven and eight positioned lower than rest of abdomen, sternite 7 reduced to simple ventral plate, tergite and sternite 8 rectangular in lateral view but tergite 8 distinctly smaller. *Hypopygium* with cercus and epandrium subrectangular, both well-developed, large surstylus articulated or fused with epandrium with specific shape, phallus filamentous, long to very long, hypandrium reduced but distinct and sickle-shaped. **Female.** Similar to male except for following characters: eyes dichoptic with lower ommatidia enlarged, frons as wide as face, legs with setae distinctly finer and shorter, base of abdomen blackish, tip pointed.

**Remarks:** *Rhamphempis* **gen. nov**. comprises four species from French Guiana and one species from the USA, Maryland. Additionally, we have specimens of this new genus from Brazil, representing at least 10 new species that will be described in an upcoming paper.

It should be noted that the absence of a radial fork in *Rhamphempis* **gen. nov**. probably has no particular phylogenetic significance in terms of affinities with other groups of Empidinae, as some species groups without a radial fork can be closely related to species groups with a radial fork (e.g., see Chvála and Pont (2014) [25]). This character is therefore only a diagnostic character, which allows a rapid recognition of the new genus when it is combined with the apomorphies found in the proboscis, wing, and male postabdomen (see the diagnosis above).

**Figure 2:**
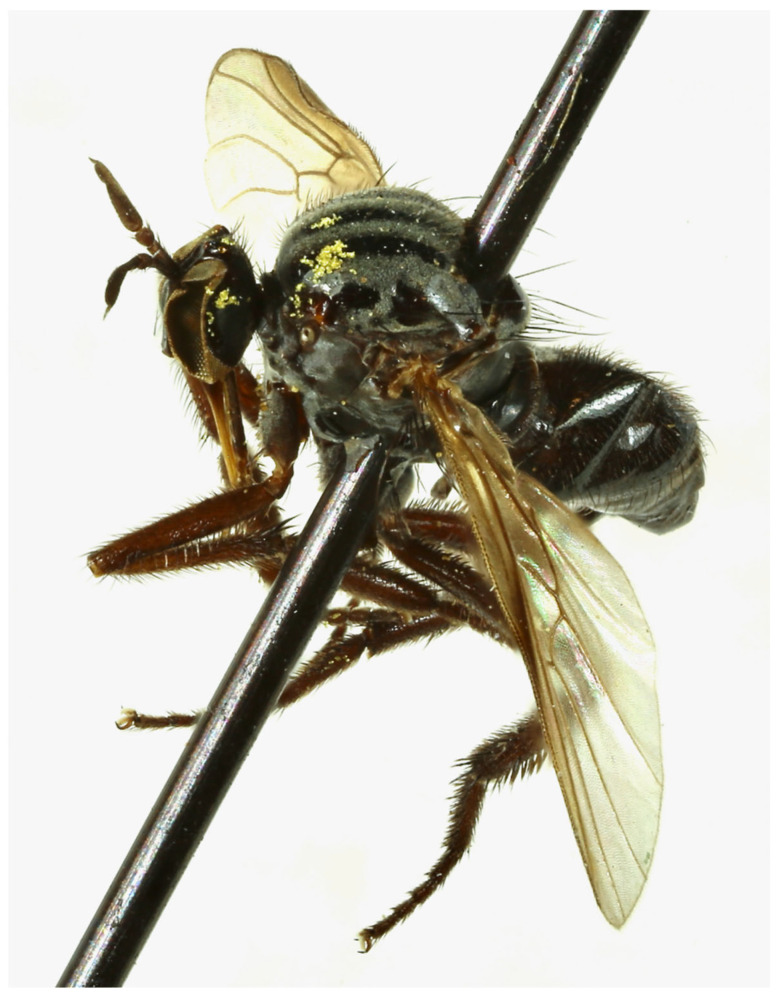
*Rhamphempis septentrionalis* **sp. nov.**, female specimen with pollen grains mainly on scutum and occiput.

The characteristic structure and shape of the proboscis (thin, long, highly sclerotised) suggest that species of *Rhamphempis* **gen. nov**. are active flower visitors, feeding on nectar. Specimens used to describe the North American species were preserved dry, and the presence of pollen grains on the body of several of them confirms this hypothesis (Figure 2).

### 3.2. Key to Species of Rhamphempis ***gen. nov.***

1.Male2- Female62.Hind trochanter with three long spines***R. montreuili*** **sp. nov.**- Hind trochanter without spines33.Thorax with distinctive pattern dorsally: scutum dusted grey to shiny blackish on acrostichals, dorsocentrals, postpronotum, postalar callus, and supra-alar area***R. septentrionalis*** **sp. nov.**- Thorax with another pattern44.Prothorax yellow, fore and mid legs mainly yellow, hind femur yellowish basally and apically, hind tibia yellow basally, rather large species (wing length about 4.5 mm)***R. concava*** **sp. nov.** Prothorax blackish to brownish, legs mainly blackish brown, smaller species (wing length about3.5 mm)55.Legs entirely blackish-brownish, wing brownish***R. distincta*** **sp. nov.** Legs brownish with tibiae somewhat yellowish at base, wing rather clear, postalar calus yellowish***R. mirifica*** **sp. nov.**6.Legs without pennation***R. montreuili*** **sp. nov.** All femora, mid, and hind tibiae with pennation***R. septentrionalis*** **sp. nov.**

### 3.3. Rhamphempis septentrionalis ***sp. nov.***

**Figure 3:**
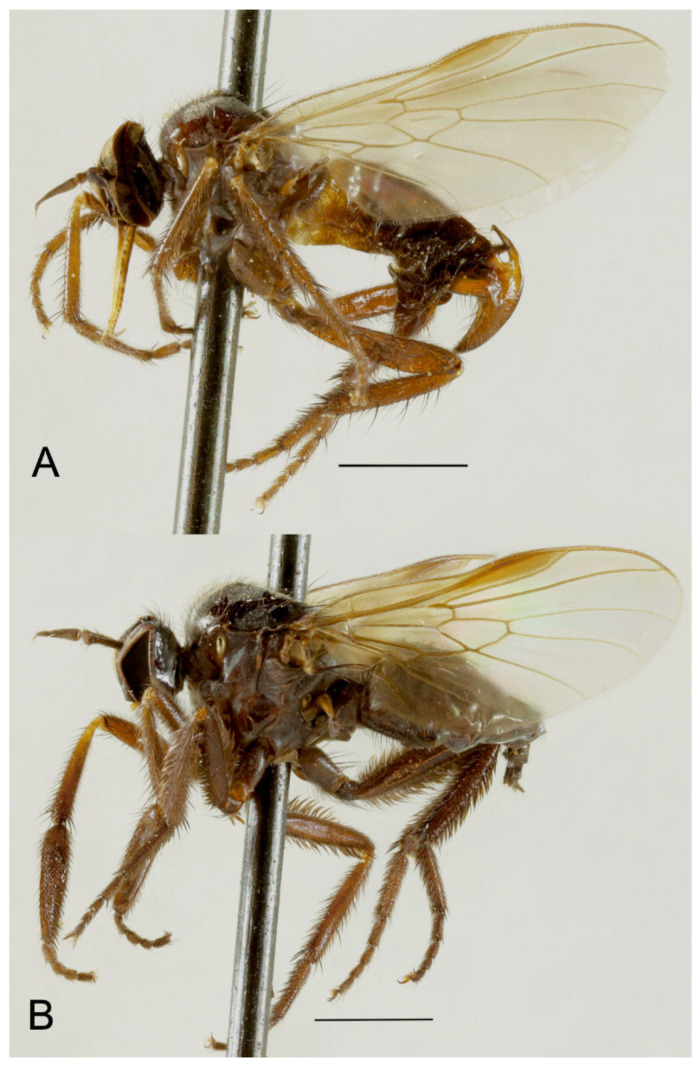
*Rhamphempis septentrionalis* **sp. nov.** (**A**) Male habitus; (**B**) female habitus. Scale bare = 1 mm.

**Figure 4:**
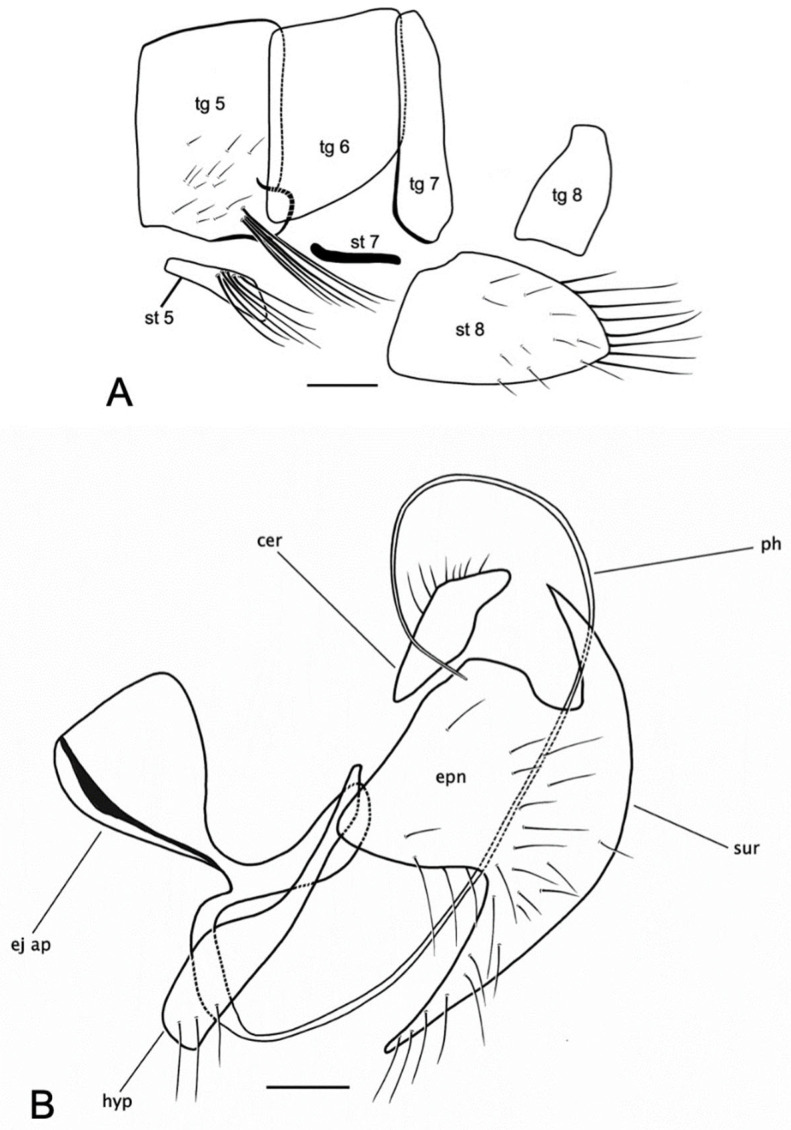
*Rhamphempis septentrionalis* **sp. nov.** (**A**) Male postabdomen; (**B**) male hypopygium. Scale bar = 0.2 mm. Abbreviations: tg—tergite; st—sternite; cer—cercus; epn—epandrium; ej ap—ejaculatory apodeme; hyp—hypandrium; ph—phallus; sur—surstylus.

(Figure 2, Figure 3A,B and Figure 4A,B)

urn:lsid:zoobank.org:act:341367E7-41D3-4A8A-8D92-D33ADBB38576

**Type material: Holotype** male, [USA]: “Maryland, College Park, 17.vi.1934, C.T. Greene collector” [38°59′22.90″ N 76°56′15.93″ W] [USNM]. **Paratypes:** two males and fourteen females, same data as holotype; three males and three females, same data as holotype except “ED11765 to ED11770” [MNHN]; two males and two females, same data as holotype except [INPA]; seven males and seven females, same data except “4.vii.1935”; four females, same data except “22.vi.1934”; one male and one female, same data except “7.viii.1938”; one male, same data except “1.viii.1931”; four females, same data except “26.vi.1932”; and one male and three females, same data except “23.vii.1933” [USNM].

**Other materials:** One male without abdomen, same data except “17.vi.1934”; and one male with part of abdomen missing, same data except “23.vii.1933” [USNM].

**Diagnosis:** Blackish species; postpedicel lengthened; scutum with a characteristic shiny pattern on a dusted grey background, scutellum with four setae in male, about eight in female; male abdomen blackish to clear brown at base, entirely black in female; and female first fore tarsomere long, swollen, female legs with distinct pennation.

**Description: Male.** *Head*: Occiput blackish with row of distinct postocular setae. Ocellar triangle prominent with pair of distinct rather short, fine setae. Frons reduced to very small triangle below ocellar triangle and above antennae. Eyes holoptic with upper ommatidia slightly larger. Scape and pedicel dark brown, postpedicel black. Scape slightly longer than pedicel, postpedicel lengthened (five times longer than pedicel); stylus short; scape and pedicel with fine, short setae. Labrum slightly longer than head height, yellowish brown; labium blackish with labella as long as prementum, distinctly annulated, strongly sclerotised, with a few minute, almost not visible hairs; palpus small, brownish with a few fine, short setae. *Thorax* brownish to blackish with dusted grey scutum showing characteristic shiny pattern on acrostichals, dorsocentrals, postpronotum, postalar callus, and supra-alar area; shiny stripes on dorsocentrals enlarged anteriorly; pleurae dusted greyish except katepisternum showing shiny brown spot. All setae black. Antepronotum with 4–5 distinct, not very long lateral setae. Postpronotum with one strong rather long seta, several fine, short anteriors. Proepisternum with one distinct seta. Prosternum with one distinct seta and a few other finer, shorter anteriors. Acrostichals irregularly biserial, short, fine. Dorsocentrals irregularly biserial, short, fine, becoming strong, long posteriorly ending in one strong, very long seta in prescutellar depression. Other strong, long setae as follows: one pre-, two stronger, longer postsutural supra-alars, one postalar, three notopleurals. Laterotergite with fan of strong, very long setae especially anteriorly. Scutellum shiny brown dorsally to dusted grey at margin, with four setae, subscutellum dusted grey. *Wing* (length = 3.5 mm) with strong, long costal seta, rather clear. Veins not undulating. Distinct brown pterostigma. Discal cell short, not truncate. A_1_ abbreviated well before margin. Halter with brownish to yellowish base and stem, blackish knob. *Legs* brownish, fore tibia almost yellowish basally. Fore femur with antero- and posteroventral row of fine, not long setae; fore tibia with a few distinct setae posterodorsally and at apical tip; fore tarsomeres 1–3 with distinct ventral setae at apical tip. Mid femur covered with fine dorsal and ventral setae, stronger, longer ventrals basally; mid tibia with anterodorsal row of five strong, long setae, dorsal row of strong, long setae, two pairs of strong, long ventrals (at middle and at apical half); first mid tarsomere with short spine-like ventral seta. Hind femur with posteroventral row of strong, long setae, covered with fine ventroanterior setae, and a posterodorsal row of finer, shorter setae; hind tibia with antero- and posterodorsal row of five strong, long setae; first hind tarsomere with a few short spine-like setae. *Abdomen* blackish except for brownish base, covered with distinct not very long black setae. *Hypopygium* distinctly clearer than remaining abdomen; epandium entirely fused with surstylus, brownish to dark yellowish, ventrally and dorsally sickle-shaped, with distinct setae especially ventrally, cercus with a few dorsal bristly hairs, hypandrium reduced to simple ventral plate, phallus fine, long, forming loop apically.

**Female:** Similar to male except for following characters: scape more distinctly longer than pedicel (about twice pedicel length), eyes dichoptic with lower ommatidia enlarged, frons as wide as face, about eight scutellar setae, wing somewhat enlarged with larger pterostigma, abdomen subshiny blackish including basally, covered with short bristly hairs except distinct dorsal and ventral setae on second segment, first fore tarsomere elongated, longer than fore tibia, swollen, fore femur with short ventral pennation, a few dorsal subpennate setae, mid femur with ventral and dorsal (except apically) pennation, mid tibia with dorsal short subpennate setae, hind femur with dorsal and ventral pennation not longer than femur depth, hind tibia with dorsal pennation except at base, not longer than tibia depth, and ventral pennation at basal third.

**Etymology:** The name refers to the geographic distribution of the species (*septentrionalis* = *from the north*).

**Distribution:** Nearctic region: USA, Maryland.

**Figure 5:**
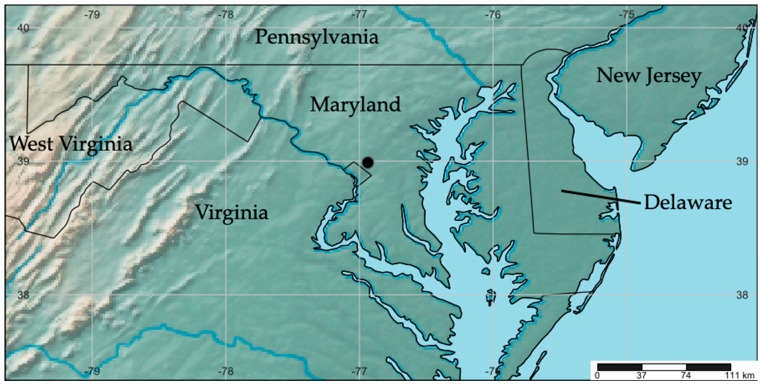
Map of eastern USA showing the distribution of *Rhamphempis septentrionalis* **sp. nov.** (black circle).

**Remarks:** At present, *R. septentrionalis* is the only species of the genus known in North America (Figure 5). It can be easily distinguished from all other species by the following combination of characters: scutum with a characteristic shiny pattern on a dusted grey background, the abbreviated A_1_, the shape of the male hypopygium, and twice as many scutellar setae in female as in male.

### 3.4. Rhamphempis concava ***sp. nov.***

**Figure 6:**
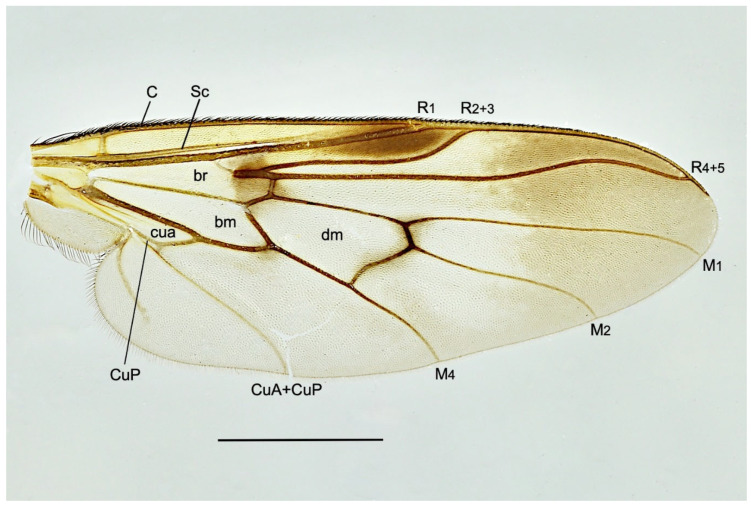
*R. concava* **sp. nov.**, wing. Abbreviations: veins: C—costa; CuA—anterior branch of cubitus; CuP—posterior branch of cubitus; M—media; R—radius; Sc—subcosta. Cells: bm—basal medial; br—basal radial; cua—anterior cubital; dm—discal medial. Scale = 1 mm.

**Figure 7:**
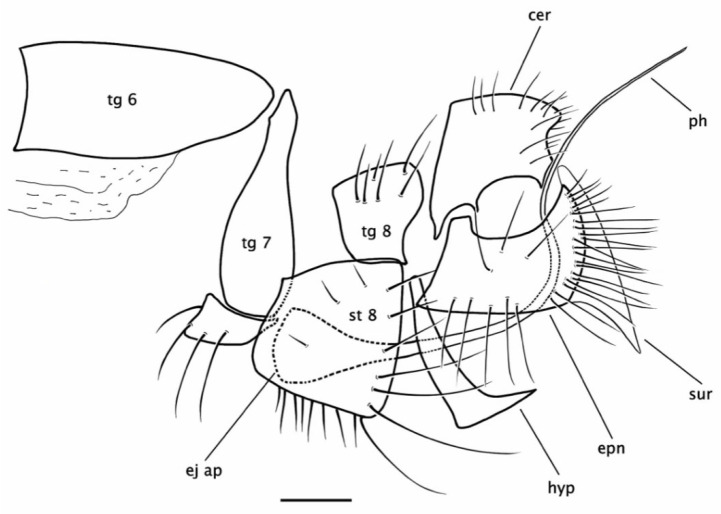
*Rhamphempis concava* **sp. nov.** Male hypopygium. Scale bar = 0.2 mm. Abbreviations: tg—tergite; st—sternite; cer—cercus; epn—epandrium; ej ap—ejaculatory apodeme; hyp—hypandrium; ph—phallus; sur—surstylus.

(Figure 6 and Figure 7)

urn:lsid:zoobank.org:act:C28FB000-71D7-459A-BDCF-735DA9A45549

**Type material: Holotype** male: “FRANCE: French Guiana, Roura, Montagne des chevaux, 6.i.2018, malaise trap, leg SEAG, ED11771” [4°43′22.8″ N 52°24′43.199″ W] [MNHN]. **Paratype**: one male, same data as holotype but “ED11772”.

**Diagnosis:** Species brownish in ground colour, scape, pedicel, fore and mid legs and prothorax mainly yellowish; hind femur yellow basally and apically, hind tibia yellow basally; scape and postpedicel lengthened, eyes holoptic with all ommatidia about the same size; R_4+5_ and M_2_ undulating; phallus characteristically S-shaped.

**Description: Male.** *Head* somewhat elongated. Occiput with row of distinct black postocular setae. Scape and pedicel yellowish brown, postpedicel blackish, scape and postpedicel elongated, about three and six times the pedicel length, respectively, stylus short. Face black, bare. Labrum yellowish brown, about twice head height; labium yellowish brown, strongly sclerotised, bare; labella as long as prementum with six distinct annulations; palpus yellowish, short with a few fine, short setae. Eyes holoptic with very narrow space between them, all ommatidia about same size. *Thorax:* Prothorax yellowish, postpronotum and postalar callus dark brown, scutum blackish, remaining brownish, distinct pair of greyish stripes (view from behind) between acrostichals and dorsocentrals. Antepronotum with a few distinct setae on each side. Postpronotum with one distinct seta and several finer, shorter anteriors. Proepisternum with 1–2 short setae, prosternum with 5–8 setae, 1–2 of which stronger, longer. Acrostichals fine, short, apparently biserial, dorsocentrals apparently irregularly biserial, ending with one strong, long seta in prescutellar depression. Three strong, long notopleurals. One strong, long postalar. Scutellum with two pairs of strong setae. Laterotergite with fan of very strong, long black setae. *Legs:* Fore coxa and femur, mid femur and tibia, hind coxa yellowish; fore tibia yellowish to somewhat brownish apically; mid coxa brownish; all tarsi yellowish except for blackish fifth tarsomere of each leg; hind femur brownish to yellow basally and apically; hind tibia brownish to yellow basally; all trochanters yellowish. Fore femur with fine anterior and posterior setae; fore tibia with antero- and posterodorsal rows of distinct, not longer than tibia depth, setae; fore tarsus with distinct setae apically except first tarsomere with a few short setae. Mid femur with fine setae; mid tibia with one dorsal row of four strong, about as long as tibia depth, setae, two strong, long setae on apical half; mid tarsomeres with distinct setae apically. Hind femur with 5–8 long posterodorsal setae, covered with many dorsal and ventral fine, rather short setae; hind tibia with antero- and posterodorsal rows of strong setae, about five strong ventral setae at apical half, and several setae apically and posteriorly; first hind tarsomere with strong ventral setae; remaining tarsomeres with distinct setae apically. All claws brownish black. *Wing* (length = 4.5 mm) spotted with distinct brown pterostigma, brownish patch around r-m and at apex of discal cell and apical quarter of wing brown. R_2+3_ characteristically curved at pterostigma level, R_4+5_, and M_2_ undulating. A_1_ complete. *Abdomen* yellow at base with many distinct setae; tergite two yellowish brown, remaining abdomen brownish. Tergites and sternites with distinct marginal setae on sides. Sternite 6 entirely membranous indistinguishable. Tergite 7 elongated downwards without distinct setae; sternite 7 reduced with 4–5 strong, long marginal setae on each side. Sternite 8 well-developed with a few strong, long posterior setae. Tergite 8 with a few dorsal setae. *Hypopygium:* Cercus large, subrectangular with ventral margin divided into two concave parts, with a few fine, short dorsal and posterior setae. Epandrium subrectangular with dorsal margin concave, many strong, long posterior and ventral setae, posteriorly prolonged by well-developed surstylus ending, pointed posteroventrally. Hypandrium somewhat reduced, posteriorly pointed in lateral view. Phallus long, thin, characteristically S-shaped.

**Female** unknown.

**Etymology:** The name refers to the characteristic concave shape of the ventral margin of the male cercus and the dorsal margin of the epandrium.

**Distribution:** Neotropical region: French Guiana.

**Remarks:** *Rhamphempis concava* can be distinguished from all other species by the following combination of characters: the yellowish colour of the prothorax and the fore and mid legs, the spotted wing and some undulating veins, as well as the characteristic S-shaped phallus.

### 3.5. Rhamphempis distincta ***sp. nov.***

(Figure 8)

urn:lsid:zoobank.org:act:1B8C618F-D20A-42A2-B4BA-24FDF5C6F09D

**Type material: Holotype** male: “FRANCE: French Guiana, Roura, Montagne des chevaux, 6.i.2018, malaise trap, leg SEAG, ED11773” [4°43′22.8″ N 52°24′43.199″ W] [MNHN].

**Diagnosis**: Blackish to brownish species of rather small size (wing length = 3.5 mm) with base of abdomen yellowish, all setae black. Scape only a little longer than pedicel; legs dark brown; surstylus characteristically lengthened and tapered ventrally; phallus thin, very long.

**Description: Male.** *Head:* Occiput blackish with row of rather strong, long black postocular setae. Ocellar triangle prominent with pair of setae. Antennae blackish, scape a little longer than pedicel, postpedicel lengthened, four times pedicel length, stylus short; scape and pedicel with short bristly hairs, postpedicel bare. Frons reduced to two small triangles below and above ocellar triangle and antennae, respectively. Face blackish, bare. Labrum long, more than 2.5 times the head height; labium strongly sclerotised, labella with a few bristly hairs, prementum with five distinct annulations; palpus brownish, labrum + labium yellowish to brownish at base. Eyes holoptic with upper ommatidia a little enlarged. *Thorax* mainly blackish but antepronotum, postpronotum, postalar calus, katepisternum, katepimeron, anepimeron brownish. Antepronotum with about six distinct short setae on each side. Proepisternum with two distinct setae. Prosternum with two strong rather long setae. Postpronotum with one strong, long basal seta and several short, fine anteriors. Remaining strong, long setae as follows: three notopleurals, one pre- and one postsutural supra-alar, one postalar, two scutellars. Most of acrostichals and dorsocentrals missing but dorsocentrals ending in two strong, long setae in prescutellar depression. Laterotergite with fan of strong, long black setae. *Legs:* All legs dark brown. Fore leg with rather fine, short setae except for a few stronger setae on tibia dorsoapically, first fore tarsomere ventrally and at apical tip, on second fore tarsomere at apical tip. Mid femur with ventral row of distinct setae, mid tibia with three strong, not longer than tibia depth, antero- and posterodorsal setae. Mid tarsomeres 1–3 with distinct strong setae ventrally and apically. Hind femur somewhat arched with antero- and posteroventral and anterodorsal rows of fine setae; hind tibia with antero- and posterodorsal rows of four strong, long setae; hind tarsus same as mid tarsus. *Wing* (length = 3.5 mm) brownish with distinct brown pterostigma. A_1_ complete. *Abdomen:* First two segments yellowish. Remaining segments brownish. Tergites 1–2 bearing strong, long marginal setae. Tergites 3–5 with distinct marginal setae. Tergite 5 with small posterolateral protuberance on each side. Sternite 5 with a few strong, long marginal setae. Sternite 6 mainly membranous, very reduced to small ventral plate. *Hypopygium:* Cercus and epandrium subrectangular, latter with small anterodorsally indentation, hypandrium short, surstylus lengthened and tapered ventrally, phallus thin, very long.

**Female:** Unknown.

**Etymology:** The name refers to a set of characters (the length of the phallus, the brownish legs and wing, and the characteristic shape of the surstylus) that clearly distinguish this species from all other *Rhamphempis* species.

**Distribution:** Neotropical region: French Guiana.

**Remarks:** *Rhamphempis distincta* can be distinguished from all other species of the genus by the combination of the following characters: scape only a little longer than pedicel, legs entirely brownish, surstylus characteristically lengthened and tapered ventrally, phallus very long.

### 3.6. Rhamphempis mirifica ***sp. nov.***

(Figure 9)

urn:lsid:zoobank.org:act:5E5D6A0F-9A3F-4A6F-9884-7653D4A32155

**Type material: Holotype** male: “FRANCE: French Guiana, Régina, Kaw, i.2003, ED11774” [4°30′54″ N 52°3′57.6″ W] [MNHN].

**Diagnosis:** Brownish species of rather small size. Labrum twice head height. Scape distinctly lengthened; legs brownish with all tibiae more or less yellowish basally; wing with brownish patch around r-m, R_2+3_ curved at pterostigma and M_2_ undulating; hypandrium sickle-shaped, phallus incredibly long.

**Description: Male.** *Head* somewhat elongated. Occiput blackish with row of postocular and occipital setae. Ocellar triangle prominent with pair of setae. Scape and pedicel brownish with a few short setae; postpedicel blackish; scape and postpedicel three and five times the pedicel length, respectively, style short. Palpus brownish with 4–5 short, fine ventral setae. Frons reduced to small blackish triangle above antennae. Face blackish, bare. Labrum long, more than 2.5 times the head height; labium brownish basally to yellowish apically; prementum with three strong annulations at base; palpus brownish, labrum + labium yellowish to brownish at base. Eyes holoptic with upper ommatidia distinctly enlarged. *Thorax* brownish with cervical sclerite, antepronotum, postpronotum, and postalar callus clearer. Most setae missing, the number of setae given when insertions are well-visible. Proepisterum and prosternum with a few distinct setae. Antepronotum with 5–6 strong, rather short setae laterally. Strong, long setae as follows: three notopleurals, one pre- and one postsutural supra-alar, one basal postpronotal, one postalar, one pair of apical and subapical scutellars, two dc in prescutellar depression. Laterotergite with fan of strong, long setae. *Legs* brownish. Fore tibia yellowish at basal half, mid and hind tibia yellowish at base. All femora with fine, short setae. First fore tarsomere with row of ventral and posteroventral short spine-like setae, second and third fore tarsomeres with spine-like setae at tip. Mid tibia with a few distinct short rather strong ventral setae at apical half, a few shorter than tibia depth distinct dorsal setae; first mid tarsomere with about 15 spine-like short ventral setae; second and third mid tarsomeres like second and third fore tarsomeres. Hind tibia with a few short distinct dorsals, one distinct short seta at apical third and a few distinct setae at tip; hind tarsus like mid tarsus but setae shorter than tarsus depth. *Wing* (length = 3.6 mm) rather clear with distinct brown pterostigma, brownish patch around r-m, R_2+3_ curved at pterostigma, M_2_ distinctly undulating, A_1_ feebly sclerotised but complete. Strong, long costal seta at base. Halter entirely yellowish to whitish. *Abdomen*: First two segments yellow, remaining segments brownish. First tergite with strong, long marginal setae on sides. Tergites 2–4 with distinct marginal setae; sternite 6 reduced but sclerotised, not entirely membranous. Sternites 7–8 with strong, long ventral setae; tergite 8 with distinct anterodorsal projection. *Hypopygium:* Cercus subtriangular. Epandrium subrectangular, with concave indentation anterodorsally, strong, long dorsal and posterior setae, extended by well-developed, apically pointed surstylus. Hypandrium somewhat sickle-shaped. Phallus thin, incredibly long, wrapped around itself apically, like a ball of wool (in studied specimen).

**Female:** Unknown.

**Etymology:** The name refers to the length and shape of the phallus that is extraordinary.

**Distribution:** Neotropical region: French Guiana.

**Remarks:** *Rhamphempis mirifica* can be distinguished from all other species by the combination of the following characters: scape distinctly lengthened, legs brownish with all tibiae more or less yellowish basally, wing with brownish patch around r-m, M_2_ undulating, phallus incredibly long.

### 3.7. Rhamphempis montreuili ***sp. nov.***

**Figure 10:**
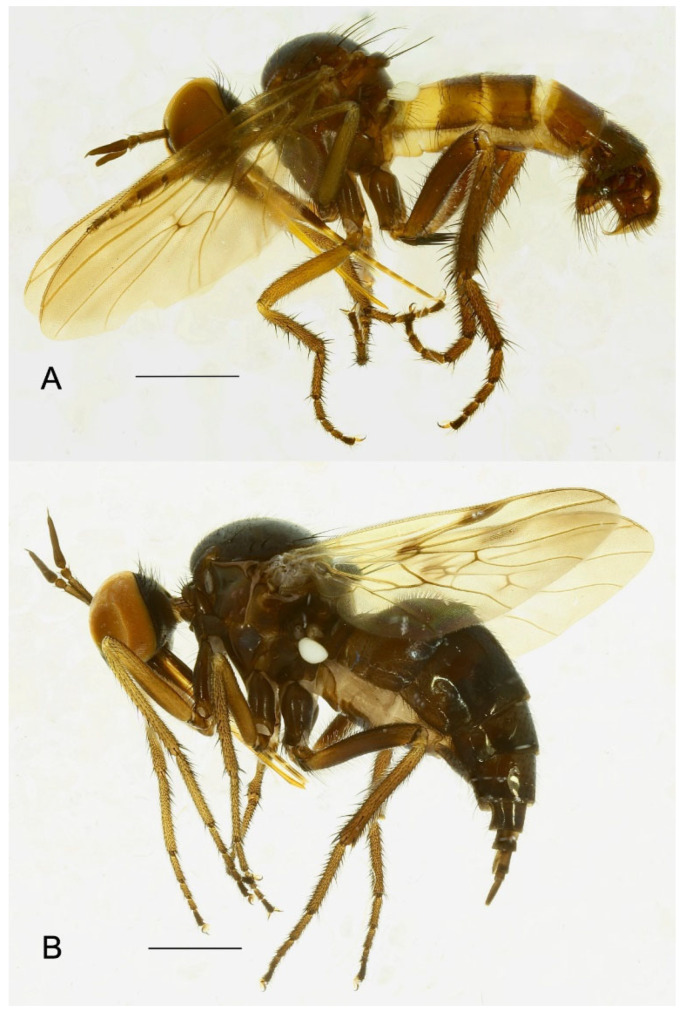
*Rhamphempis montreuili* **sp. nov.** (**A**) Male habitus; (**B**) female habitus (specimens in alcohol). Scale bar = 1 mm.

**Figure 11:**
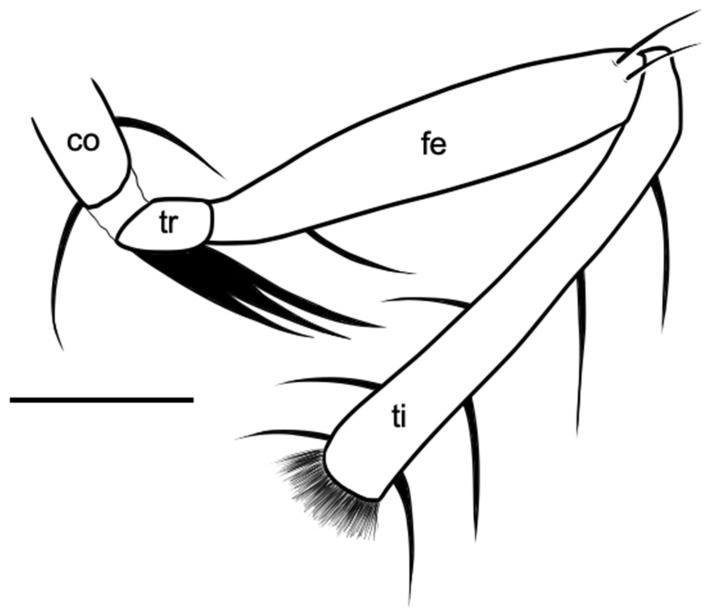
*Rhamphempis montreuili* **sp. nov.** Male hind leg (tarsus omitted) showing the characteristic spines on the trochanter, lateral view. Scale bar = 0.5 mm. Abbreviations: co—coxa; fe—femur; ti—tibia; tr—trochanter.

**Figure 12:**
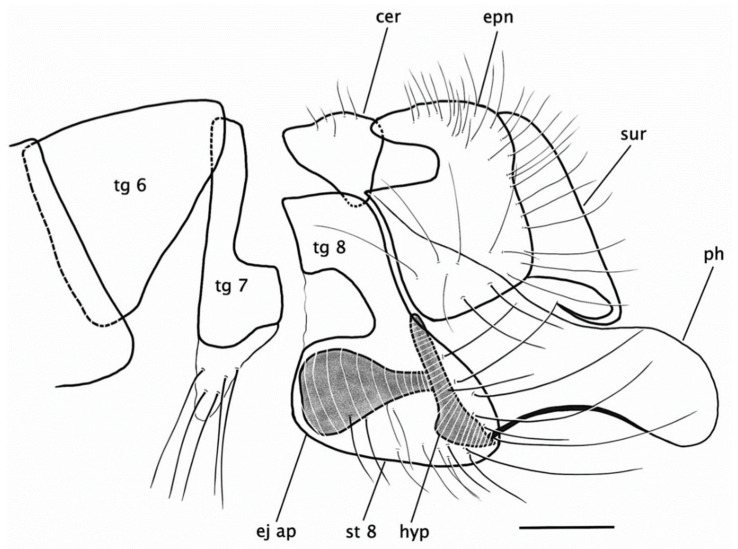
*Rhamphempis montreuili* **sp. nov.** Male hypopygium. Scale bar = 0.2 mm. Abbreviations: tg—tergite; st—sternite; cer—cercus; epn—epandrium; ej ap—ejaculatory apodeme; hyp—hypandrium; ph—phallus; sur—surstylus.

(Figure 1, Figure 10A,B, Figure 11 and Figure 12)

urn:lsid:zoobank.org:act:E7D55ACE-257D-440F-B41C-B07336BE8EA3

**Type material: Holotype** male: “FRANCE: French Guiana, Saint-Georges-de-l’Oyapock (primary rain forest), 4°04.45′ N 52°07.141′ W, 8.ii.2018, malaise trap, leg. Olivier Montreuil, ED11775” [MNHN]. **Paratypes:** one male, same data as holotype but “ED11776”; one male, “FRANCE: French Guiana, Roura, Montagne des chevaux, 6.i.2018, malaise trap, leg SEAG” [INPA]; one male, same data as previous specimen but “ED11777” [MNHN]; one female, “FRANCE: French Guiana, Saint-Georges-de-l’Oyapock, malaise trap G06, 4°04.268′ N 052°02.650′ W, 64 m, 24-28.iii.2022, C. Daugeron, ED11779” [MNHN]; one female, same data as preceding but “ED11780”.

**Other materials:** Two specimens are not in very good condition. One male, “FRANCE: French Guiana, Mitaraka, near MIT-A-RBF1 (2°14′11.4″ N 54°27′07.0″ W), 6 m, 24.iii.2015, malaise trap (289/Mita/193), leg. J. Touroult & E. Poirier”; one female, same data as holotype.

**Diagnosis:** Blackish species with base of abdomen and base of hind femur and tibia yellowish; scape lengthened; male with three long spines on hind trochanter, hind femur and tibia somewhat swollen, surstylus characteristically recurved anteroventrally.

**Description: Male.** *Head:* Occiput blackish with row of distinct black postocular setae. Scape and postpedicel lengthened, 3 and 3.5 times the pedicel length, respectively, stylus short. Face blackish, bare. Labrum yellowish to blackish brown at base, about 2.5 times head height; labium fine, strongly sclerotised, bare, blackish brown with labella longer than prementum with six distinct annulations; palpus blackish with a few fine setae. Eyes holoptic with a very narrow space between them, upper ommatidia enlarged. *Thorax* blackish to brownish laterally. Antepronotum with ring of distinct setae. Postpronotum with one strong, long basal seta and about ten finer anteriors. Proepisternum with distinct seta, prosternum with one strong, long seta. Acrostichals and dorsocentrals fine, short, irregularly biserial, latter ending with two strong, long setae in prescutellar depression; other strong, long setae as follows: three notopleurals, one pre- and one postsutural supra-alar, one postalar, one pair of apical and subapical scutellars. Laterotergite with fan of very strong, long setae. *Legs:* All coxae blackish brown. Fore femur yellowish, without distinct setae; fore tibia yellowish with a few distinct dorsal setae not longer than tibia depth; fore tarsus dark yellowish but fifth tarsomere blackish, first tarsomere with rather strong, short ventral setae, remaining tarsomeres with distinct apical circlet of setae, claws yellowish at base, blackish apically. Mid femur brownish to yellowish with short, fine dorsal setae; mid tibia yellowish with short, rather strong dorsal and ventral setae; mid tarsus similar to fore tarsus. Hind trochanter yellowish with three long posteroventral spines projected backwards; hind femur dark brown to almost yellowish basally, somewhat swollen, with a few distinct fine dorsal setae; hind tibia dark brown to yellowish basally, somewhat swollen, with 4–5 and 2–3 distinct dorsal and apicoventral setae, respectively, almost as long as tibia depth; hind tarsus dark brown blackish with many strong, as long as tarsus depth, ventral and dorsal setae. *Wing* (length = about 3.5 mm) brown with very distinct brown pterostigma, brown patch at intersection of veins r-m, R_4+5_, R_2+3_, and base of discal cell; R_2+3_ characteristically curved at pterostigma; R_4+5_ unforked, M_2_ somewhat undulating; A_1_ complete; C ends at R_4+5_ with a distinct seta at base. Halter yellowish to whitish. *Abdomen* mainly brownish to dark brown to yellowish at base. Sternite 6 entirely membranous, indistinguishable. Segment seven considerably modified: tergite 7 elongated downwards, partially fused with sternite 7; sternite 7 with a ring of strong, long setae. Sternite 8 well-developed posteriorly with about 15 strong, long ventral and posterior setae. *Hypopygium:* Cercus rather large, subtriangular with a few fine, short dorsal setae. Epandrium subrectangular, characteristically recurved anterodorsally, with about 15 long posterior and ventral setae and many shorter dorsals, posteriorly prolonged by a well-developed surstylus ending in a pointed anteroventrally recurved part. Hypandrium somewhat reduced. Phallus long, thin.

**Female**: Similar to male except for the following characters: dichoptic, frons blackish, all ommatidia of equal size, setae on legs distinctly finer, shorter, trochanter without spines, M_2_ more distinctly undulating, tergite 3 densely covered with short, fine dorsal and lateroventral setae, tip of abdomen pointed.

**Etymology:** The species is dedicated to one of the collectors, Olivier Montreuil.

**Distribution:** Neotropical region: French Guiana.

**Remarks:** *R. montreuili* can be distinguished from all other species by the combination of the following characters: scape lengthened, base of hind femur and tibia yellowish, male hind trochanter with three long spines, phallus very thin, female legs without pennation.

## 4. Discussion

**Figure 13:**
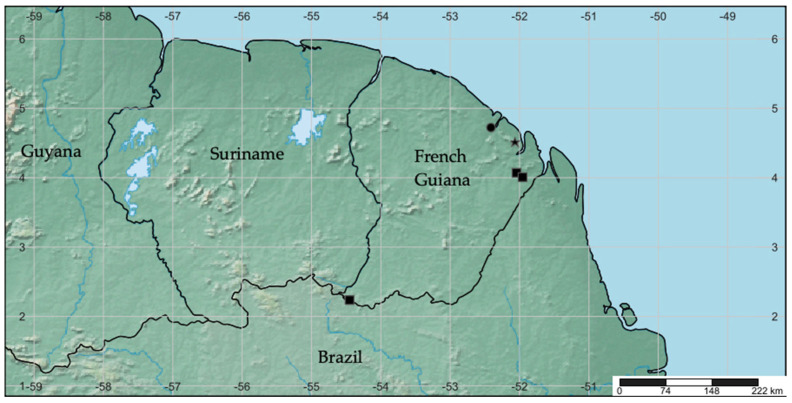
Map of French Guiana showing the distribution of *Rhamphempis concava* **sp. nov.** and *Rhamphempis distincta* **sp. nov.** (black circle), *Rhamphempis mirifica* **sp. nov.** (black star), and *Rhamphempis montreuili* **sp. nov.** (black square).

*Rhamphempis* **gen. nov.** includes five species (as ten further await description), four in the Amazonian rainforest of French Guiana (Figure 13) and one in the USA, Maryland. In spite of this outstanding disjunct geographic distribution, several morphological characters support the monophyly of *Rhamphempis*, such as the presence of spaced rings on the labium, the strong modification of the male pregenital segments, and the presence of large surstylus (a posterior extension of the epandrium), which are found both in the only known North American species and in all species discovered in French Guiana.

Maryland and French Guiana have very different climates. French Guiana has an equatorial and rather stable climate: the temperatures vary little throughout the year (around 26 °C on average), and the rainfall is high all year round, with the exception of the September–November period, when it decreases significantly but does not disappear completely. College Park, Maryland has a temperate climate: the temperatures vary considerably, from less than 5 °C in winter (December to February) to 23.5 °C on average in summer (June to September); however, College Park experiences high levels of precipitation throughout the year, including the summer months (with about 100 mm/month between June and September) (e.g., see https://climate-data.org (accessed on 8 January 2024)). So, College Park is characterized by a warm, humid summer resembling a humid subtropical climate. The distribution of *Rhamphempis* is, therefore, likely related to the warm, humid conditions available in College Park during the summer months and in French Guiana all year round.

This observed disjunction may be a collection artefact involving the presence of additional species distributed in various regions located between Maryland and the Amazonian basin and offering similar climate conditions at one time or another during the year.

The phylogenetic relationships of *Rhamphempis* within the tribe Empidini are not known. However, the shape and structure of the proboscis (thin, highly sclerotised, with minute hairs, or bare and apparently without pseudotracheae) suggest that this genus belongs to the large clade called the *Empis* group or *Empis* lineage (Daugeron & Winkler (2010) [18], Winkler et al. (2010) [19], and Watts et al. (2016) [20]), which is considerably diverse in the northern hemisphere with a number of genera, subgenera, and species groups, including hundreds of species. Consequently, *Rhamphempis* could have a northern origin, and its presence in areas with a warm, humid summer climate would have been a kind of pre-adaptation to its dispersal and diversification in tropical South America. This hypothesis will have to be tested by phylogenetic analyses in addition to the probable discovery of new species throughout the New World.

## Figures and Tables

**Figure 8:**
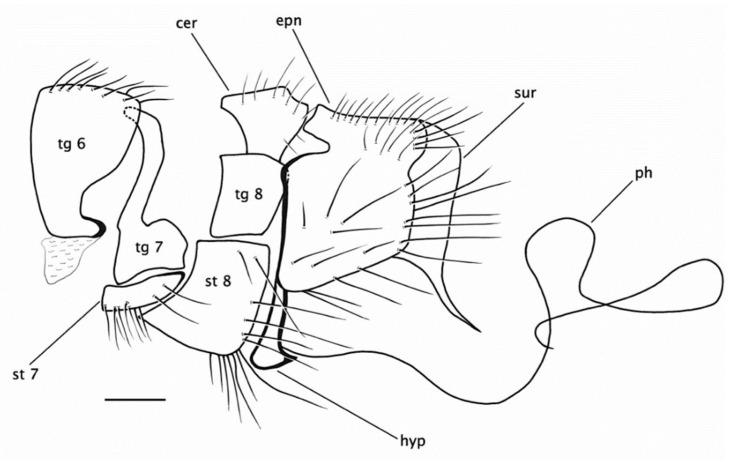
*Rhamphempis distincta* **sp. nov.** Male hypopygium. Scale bar = 0.2 mm. Abbreviations: tg—tergite; st—sternite; cer—cercus; epn—epandrium; ej ap—ejaculatory apodeme; hyp—hypandrium; ph—phallus; sur—surstylus.

**Figure 9:**
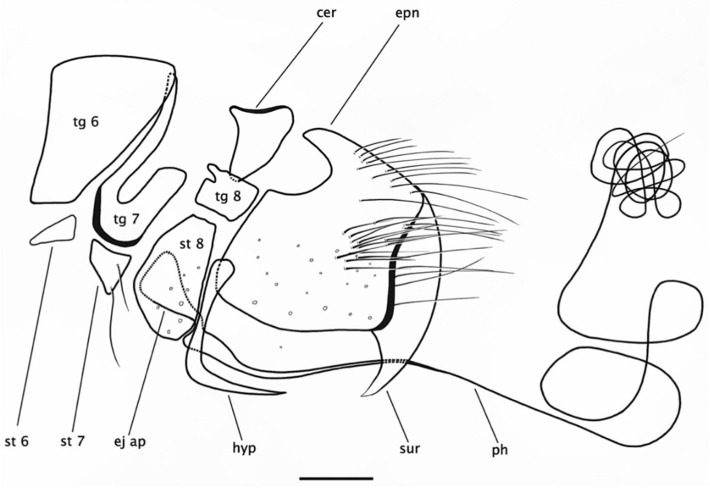
*Rhamphempis mirifica* **sp. nov.** Male hypopygium. Scale bar = 0.2 mm. Abbreviations: tg—tergite; st—sternite; cer—cercus; epn—epandrium; ej ap—ejaculatory apodeme; hyp—hypandrium; ph—phallus; sur—surstylus.

## Data Availability

Data are contained within the article.
